# Quantitative radiological analysis and clinical outcomes of urgent EC-IC bypass for hemodynamic compromised patients with acute ischemic stroke

**DOI:** 10.1038/s41598-022-12728-x

**Published:** 2022-05-25

**Authors:** Hyunjun Jo, Dongwook Seo, Young Deok Kim, Seung Pil Ban, Tackeun Kim, O-Ki Kwon, Chang Wan Oh, Leonard Sunwoo, Beom Joon Kim, Moon-Ku Han, Hee-Joon Bae, Si Un Lee, Jae Seung Bang

**Affiliations:** 1 Department of Neurosurgery, Korea University Ansan Hospital, Korea University College of Medicine, Ansan-si, Korea; 2 Department of Neurosurgery, Soonchunhyang University Seoul Hospital, Soonchunhyang University College of Medicine, Seoul, Korea; 3 Department of Neurosurgery, Seoul National University Bundang Hospital, Seoul National University College of Medicine, 82 Gumi-ro 173 beon-gil, Bundang-gu, Seongnam-si, Gyeonggi-do 13620 Korea; 4 Department of Radiology, Seoul National University Bundang Hospital, Seoul National University College of Medicine, Seongnam-si, Korea; 5 Department of Neurology, Cerebrovascular Disease Center, Seoul National University Bundang Hospital, Seoul National University College of Medicine, Seongnam-si, Korea

**Keywords:** Medical research, Neurology

## Abstract

This study aimed to demonstrate the effectiveness of urgent extracranial-to-intracranial bypass (EIB) in acute ischemic stroke (AIS) through quantitative analysis of computed tomography perfusion (CTP) results using RAPID software. We retrospectively analyzed 41 patients who underwent urgent EIB for AIS under strict operation criteria. The quantitative data from CTP images were reconstructed to analyze changes in pre- and postoperative perfusion status in terms of objective numerical values using RAPID software. Short- and long-term clinical outcomes, including complications and neurological status, were also analyzed. Postoperatively, the volume of time-to-max (Tmax) > 6 s decreased significantly; it continued to improve significantly until 6 months postoperatively (preoperative, 78 ml (median); immediate postoperative, 23 ml; postoperative 6 months, 7 ml; *p* = 0.000). Ischemic core-penumbra mismatch volumes were also significantly improved until 6 months postoperatively (preoperative, 72 ml (median); immediate postoperative, 23 ml; postoperative 6 months, 5 ml; *p* = 0.000). In addition, the patients’ neurological condition improved significantly (*p* < 0.001). Only one patient (2.3%) showed progression of infarction. Urgent EIB using strict indications can be a feasible treatment for IAT-ineligible patients with AIS due to large vessel occlusion or stenosis.

## Introduction

Treatment of cerebral infarction has been rapidly increasing in recent decades. Currently, intra-arterial thrombectomy (IAT) is the prescribed treatment for patients with large vessel occlusion cerebral infarction within 24 h after symptom onset^1,2^. In contrast, extracranial-to-intracranial (EC-IC) bypass for IAT-ineligible patients has not been extensively utilized as it did not demonstrate any benefit over medical treatment in two randomized controlled trials, the EC-IC Bypass Trial (EIBT) and the Carotid Occlusion Surgery Study (COSS)^3–5^. Nevertheless, despite recent rapid technological and instrumental advances, IAT still presents a relatively high failure probability and a contraindication of approximately 10%–20% of all cases^1,2^. Currently, in these cases, based on evidence-based medicine, the only potential treatment involves medical therapy including induced hypertension treatment and dual antiplatelet therapy. However, EC-IC bypass (EIB) has proven effective in a large number of patients^6–10^.

EIB is mainly conducted on IAT-ineligible patients and has been observed to optimize postoperative condition and prevent recurrent stroke. Nevertheless, previous studies have been limited in terms of limited sample size, short follow-up periods, and a lack of consensus regarding indications for EIB in acute ischemic stroke (AIS). Many studies have therefore been performed including on patients in the subacute phase^7,8,11^. In addition, although most studies have demonstrated clinical improvement, they have not conducted quantitative analyses of imaging improvements to produce more objective evidence. Therefore, we performed a quantitative analysis of pre- to postoperative changes in cerebral perfusion using computed tomography perfusion (CTP) with RAPID software, whose findings were considered in conjunction with clinical outcomes to determine the efficacy and safety of EIB for IAT-ineligible patients with AIS.

## Materials and methods

### Operation indication

This retrospective study was approved by our hospital’s institutional review board (B-2102/666-105). We performed urgent EIB on patients with AIS due to intracranial stenosis or occlusion who met all of the following four indications: (1) hemodynamic cerebral infarction on a computed tomography (CT) scan or magnetic resonance imaging (MRI), with perfusion delay on imaging; (2) diffusion–perfusion mismatch on examination; (3) neurological deterioration (ND) despite optimum medical treatment for cerebral infarction including antiplatelet therapy and induced hypertension; and (4) ineligibility for IAT using a stent retriever or direct aspiration due to contraindications or a failed intervention. ND was defined as the onset of new neurological symptoms, an increase in National Institutes of Health Stroke Scale (NIHSS) score by two or more points, an increase in the NIHSS consciousness score by one point or more, or an increase in the NIHSS motor score by one point or more during the patient’s hospital stay^12^. When AIS patients admitted to our neurology department showed ND despite optimum medical treatment, the risk of ongoing infarction was considered beyond the timeframe from the first infarction. If the patient was ineligible for other treatments such as IAT and met the criteria for EIB, it was performed as soon as possible. All patients in this study underwent EIB within two weeks of symptom onset.

### Surgical procedure

Under general anesthesia, the parietal or frontal branch of the superficial temporal artery (STA) was dissected and used as the donor artery. We performed craniotomy around the hypoperfused area so blood flow could be supplied to the location of perfusion delay. Nevertheless, the vessel supplying blood to the eloquent area was not bypassed considering the possibility of complications. As a result, most of the craniotomy was performed around the Chater’s point, which is likely to have blood vessels such as angular, supramarginal, and posterior temporal arteries. After an approximately 5-cm-diameter craniotomy was performed, the STA and distal MCA (M4) branch were anastomized with 8–10 stitches using prolene 10–0. Then, intraoperative STA flow was measured using an ultrasonic flow meter. As the surgery was performed on patients who could not undergo preoperative medical therapy, dual antiplatelet therapy was maintained intraoperatively, and aspirin was resumed from the day after surgery. Intensive care was administered intra- and postoperatively to avoid lowering blood pressure (> 120 mmHg). When the required blood flow was observed to be sufficiently large, a double-barrel bypass was performed.

### Radiological analysis

The CTP data of patients who underwent EIB were quantitatively reconstructed using RAPID software (Version 5.0.4, iSchemaView, Menlo Park, California, USA), which provides an automated perfusion measurement, precluding reliance on the subjective interpretation of imaging studies, and the perfusion status of these patients was quantitatively evaluated^13^. CTP was performed using a 256-slice CT scanner (Brilliance, Philips Medical Systems, Best, Netherlands). Changes in time-to-maximum (Tmax) > 10 s, > 8 s, > 6 s, and > 4 s on time-to-peak (TTP) were analyzed. Cerebral blood flow (CBF) < 30%, representing the ischemic core volume^14^, was also analyzed. A Tmax > 6 s indicates penumbra^14^; therefore, the difference between Tmax > 6 s and CBF < 30% was defined as the diffusion–perfusion mismatch volume, and this volume was also analyzed.

In our hospital, RAPID software was introduced in 2018 and allowed the reconstruction of previous CTP data from 2006 onward. Patients who underwent EIB were given a routine CTP within 24 h preoperatively, within 48 h postoperatively, and at six months postoperatively. Therefore, this study analyzed the CTP data performed at these three time points of patients who underwent urgent EIB between 2006 and 2020.

In addition, all patients underwent transfemoral cerebral angiography (TFCA) one week and six months after surgery to confirm the bypass patency.

### Clinical outcomes

To analyze the patient’s clinical results, preoperative modified Rankin Scale (mRS) and the NIHSS were used, and the results were compared with NIHSS at discharge and mRS at last follow-up, that revealed short-term and long-term outcomes, respectively. Postoperatively, mRS of ≤ 2 was defined as good outcome and ≥ 3 was defined as a bad outcome. We defined a new infarction showing no specific symptoms but accidently identified as a silent infarction. If a new infarction accompanied by symptoms was identified on the ipsilateral side of the surgery postoperatively during hospital stay, it was defined as infarction progression. The manifestation of neurological symptoms arising from the hemisphere on which surgery was performed > 6 months postoperatively, was defined as stroke recurrence, following radiological confirmation.

### Statistical analysis

Statistical analysis was performed using IBM SPSS statistics 25 (SPSS, Chicago, Illinois). Continuous variables were presented as median (interquartile ranges). The Wilcoxon matched-pairs signed-rank test was used to compare perfusion data before and after bypass, and the Friedman test was used to compare the data at three time points, including perfusion data at 6 months postoperatively. Univariate logistic regression was performed to see if there are factors predicting a good outcome. Multivariable analysis was only performed for variables with a *p* value < 0.1 after univariable analysis. Backward elimination, that is a multivariable logistic regression that removes the variables one by one in a backward fashion, was performed. In addition, to confirm if there was a significant difference in the perfusion parameters as well as other variables between the good outcome and the bad outcome groups, we used Mann–Whitney U-test for continuous variables, while chi-square test was employed for categorical variables. Generally, a *p* value of < 0.05 was considered as significant in this study.

### Statement of ethics

The current study was approved by the Institutional Review Board and Ethical Committee of Seoul National University Bundang Hospital (B-2102/666-105), and the requirement for obtaining individual patient consent was waived owing to the retrospective nature of the study. Our research was conducted in accordance with the ethical standards of the aforesaid ethics committee and the tenets of the 1964 Declaration of Helsinki and its later amendments.

## Results

### Baseline characteristics

**Figure 1:**
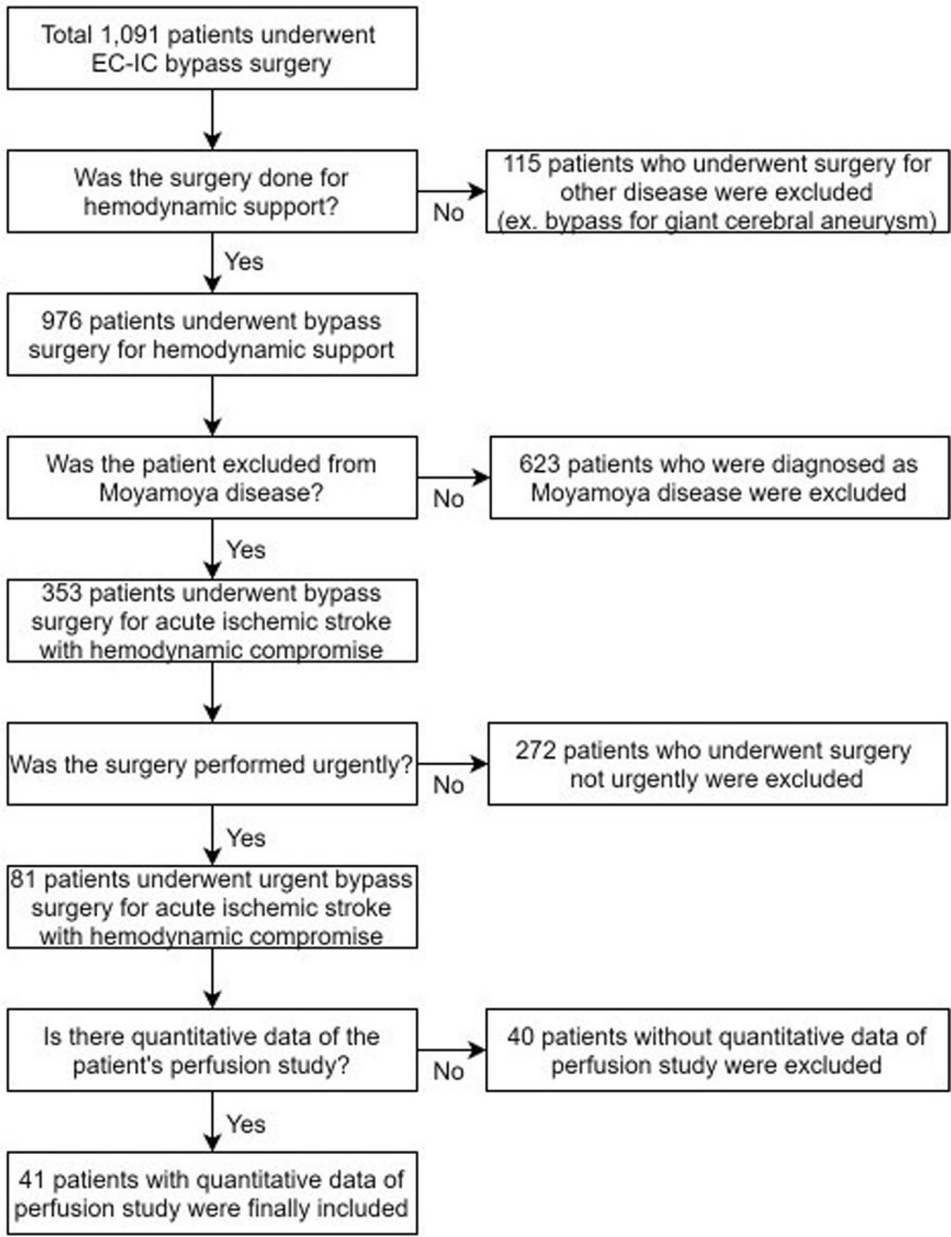
Flow diagram of the enrolled patients.

From January 2006 to December 2020, we performed urgent EIB on 79 hemispheres of 78 patients with acute ischemic stroke and intracranial arterial stenosis or occlusion with cerebral infarction.s Among them, 42 hemispheres of 41 patients had the pre- and postoperative quantitative perfusion data; only these patients were analyzed in this paper (Fig. 1). The median age of the patients was 66.5 (61.75–72.25) years, and it took 3 (0–5) days from first symptom onset to hospitalization. Neurologists classified the patients according to the Trial of Org 10172 in Acute Stroke Treatment (TOAST) classification; 35 (83.3%) patients were classified as having large-artery atherosclerosis (LAA), 3 (7.1%) as having cardioembolism (CE), and it was not clear whether they had LAA or CE in 4 (9.5%) patients. All seven patients also had large vessel atherosclerosis. Preoperative perfusion examination of the patients showed Tmax > 10 s of 13 ml (0–36.25), Tmax > 8 s of 34 ml (13.5–90.25), Tmax > 6 s of 83 ml (53.75–168.75), Tmax > 4 s of 216.5 ml (155.25–296), CBF < 30% of 0 ml (0–8.75), and mismatch volume of 74.5 ml (40.5–155.5) along with hemodynamic compromise. Moreover, 10 (23.8%) of the 42 hemispheres underwent IAT but failed, whereas the remaining 32 (76.2%) were not indicated for IAT. Of these, 22 patients were beyond the timeframe, 9 did not undergo IAT due to the high risk considering their vascular status, and 1 was not indicated for IAT because the symptoms were not severe. It took 4 (3–11.25) days from the first symptom onset to surgery. The location of the lesion, the number of anastomosis performed, and the preoperative mRS and NIHSS distribution are presented in Table 1.

**Table 1:** Patients baseline characteristics.

Variables	Total	Good outcome	Bad outcome	*p* value
Total (%)	42	27 (64.3)	15 (35.7)	
Age*	66.5 (61.75–72.25)	68 (62–73)	65 (59–70)	0.372
Sex (male (%))	29 (69.0)	18 (66.7)	11 (73.3)	0.654
Side (right (%))	17 (40.5)	11 (40.7)	6 (50.0)	0.963
**Medical history, number (%)**				
Hypertension	27 (64.3)	18 (66.7)	9 (60.0)	0.666
Diabetes mellitus	14 (33.3)	9 (33.3)	5 (33.3)	1.000
Hyperlipidemia	16 (38.1)	12 (44.4)	4 (26.7)	0.256
Onset to operation, days*	4 (3–11.25)	6 (3–10)	9 (6–13)	0.235
**TOAST classification (%)**			0.821	
LAA	35 (83.3)	23 (85.2)	12 (80.0)	
Cardioembolism	3 (7.1)	2 (7.4)	1 (8.3)	
Others	4 (9.5)	2 (7.4)	2 (13.3)	
**Location of lesion (%)**			0.094	
Proximal ICA	18 (42.9)	10 (37.0)	8 (53.3)	
Distal ICA	6 (14.3)	2 (7.4)	4 (26.7)	
M1	15 (35.7)	12 (44.4)	3 (20.0)	
M2	3 (7.1)	3 (11.1)	0 (0.0)	
**Lesion severity (%)**			0.227	
Occlusion	37 (88.1)	25 (92.6)	12 (80.0)	
Severe stenosis	5 (11.9)	2 (7.4)	3 (20.0)	
**Number of anastomosis (%)**			0.666	
Single barrel	35 (83.3)	22 (81.5)	13 (86.7)	
Double barrel	7 (16.7)	5 (18.5)	2 (13.3)	
PreOp mRS*	4 (3–4)	3 (2–4)	4 (3–4)	0.241
PreOp NIHSS*	9 (5.75–12)	9 (5–12)	9 (8–12)	0.470
PreOp RAPID*				
Tmax > 10 s	13 (0–36.25)	8 (0–34)	23 (4–60)	0.387
Tmax > 8 s	34 (13.5–90.25)	27 (6–56)	50 (19–119)	0.123
Tmax > 6 s	83 (53.75–168.75)	75 (30–120)	150 (63–209)	0.054
Tmax > 4 s	216.5 (155.25–296)	192 (132–268)	281 (179–344)	0.155
CBF < 30%	0 (0–8.75)	0 (0–12)	0 (0–8)	0.611
Mismatch volume	74.5 (40.5–155.5)	56 (13–120)	125 (63–209)	0.044
PostOp 0 RAPID*				
Tmax > 10 s	0 (0–3.5)	0 (0–0)	0 (0–10)	0.183
Tmax > 8 s	0 (0–7)	0 (0–5)	3 (0–36)	0.178
Tmax > 6 s	21 (2.25–48.75)	11 (0–35)	48 (23–100)	0.027
Tmax > 4 s	140.5 (64.75–226.75)	124 (57–157)	224 (173–259)	0.039
CBF < 30%	0 (0–0)	0 (0–0)	0 (0–0)	0.088
Mismatch volume	20.5 (0–48.75)	11 (0–35)	48 (23–100)	0.020

CBF, cerebral blood flow; CI, confidence interval; mRS, ICA, internal carotid artery; LAA, large-artery atherosclerosis; modified Rankin Scale; NIHSS, National Institute of Health Stroke Scale; OR, odds ratio; preOp, preoperative; postOp 0, immediate postoperative; s, seconds; Tmax, time-to-maximum; TOAST, Trial of Org 10172 in Acute Stroke Treatment

*Median (interquartile).

### Radiological analysis (See representative cases in Supplementary Fig. S1 and Fig. S2 online)

#### Quantitative assessment of immediate postoperative perfusion status

We quantitatively analyzed 42 patients whose data of CTP could be reconstructed with RAPID. Preoperatively and immediately postoperatively, Tmax > 10 s was 13 ml (0–36.25) and 0 ml (0–3.5) respectively, showing a statistically significant difference (*p* = 0.000). Tmax > 8 s also showed a significant difference of 34 ml (13.5–90.25) and 0 ml (0–7) preoperatively and postoperatively. Both Tmax > 6 s (83 ml [53.75–168.75] to 21 ml [2.25–48.75], *p* = 0.000) and Tmax > 4 s (216.5 ml [155.25–296] to 140.5 ml [64.75–226.75], *p* = 0.000) also improved significantly. The pre- and postoperative volume of CBF < 30% decreased from 0 ml (0–8.75) to 0 ml (0–0), which was statistically significant (*p* = 0.014). The diffusion–perfusion mismatch volume was calculated as the difference between Tmax > 6 s and CBF < 30%, and it decreased significantly from 74.5 ml (40.5–155.5) to 20.5 ml (0–48.75; *p* = 0.000) (Table 2).

**Table 2:** Short- and long-term comparison of CTP data (median (interquartile)).

Short term comparison of CTP quantitative data (n = 42)				
CTP parameter	PreOp	PostOp 0		*p* value
Tmax > 10 s (ml)	13 (0–36.25)	0 (0–3.5)		0.000
Tmax > 8 s (ml)	34 (13.5–90.25)	0 (0–7)		0.000
Tmax > 6 s (ml)	83 (53.75–168.75)	21 (2.25–48.75)		0.000
Tmax > 4 s (ml)	216.5 (155.25–296)	140.5 (64.75–226.75)		0.000
CBF < 30% (ml)	0 (0–8.75)	0 (0–0)		0.014
Mismatch volume (ml)	74.5 (40.5–155.5)	20.5 (0–48.75)		0.000

Long term comparison of CTP quantitative data (n = 31)				
CTP parameter	PreOp	PostOp 0	PostOp 6 M	*p* value
Tmax > 10 s (ml)	12 (0–34)	0 (0–6)	0 (0–0)	0.000
Tmax > 8 s (ml)	27 (15–89)	0 (0–9)	0 (0–8)	0.000
Tmax > 6 s (ml)	78 (61–159)	23 (0–65)	7 (0–22)	0.000
Tmax > 4 s (ml)	219 (164–281)	150 (79–236)	118 (43–223)	0.000
CBF < 30% (ml)	0 (0–12)	0 (0–0)	0 (0–5)	0.023
Mismatch volume (ml)	72 (47–148)	23 (0–57)	5 (0–17)	0.000

CBF, cerebral blood flow; CTP, perfusion computed tomography; preOp, preoperative; postOp 0, immediate postoperative; postOp 6 M, postoperative 6 months; Tmax, time-to-maximum;

#### Quantitative assessment of long-term postoperative perfusion status

**Figure 2:**
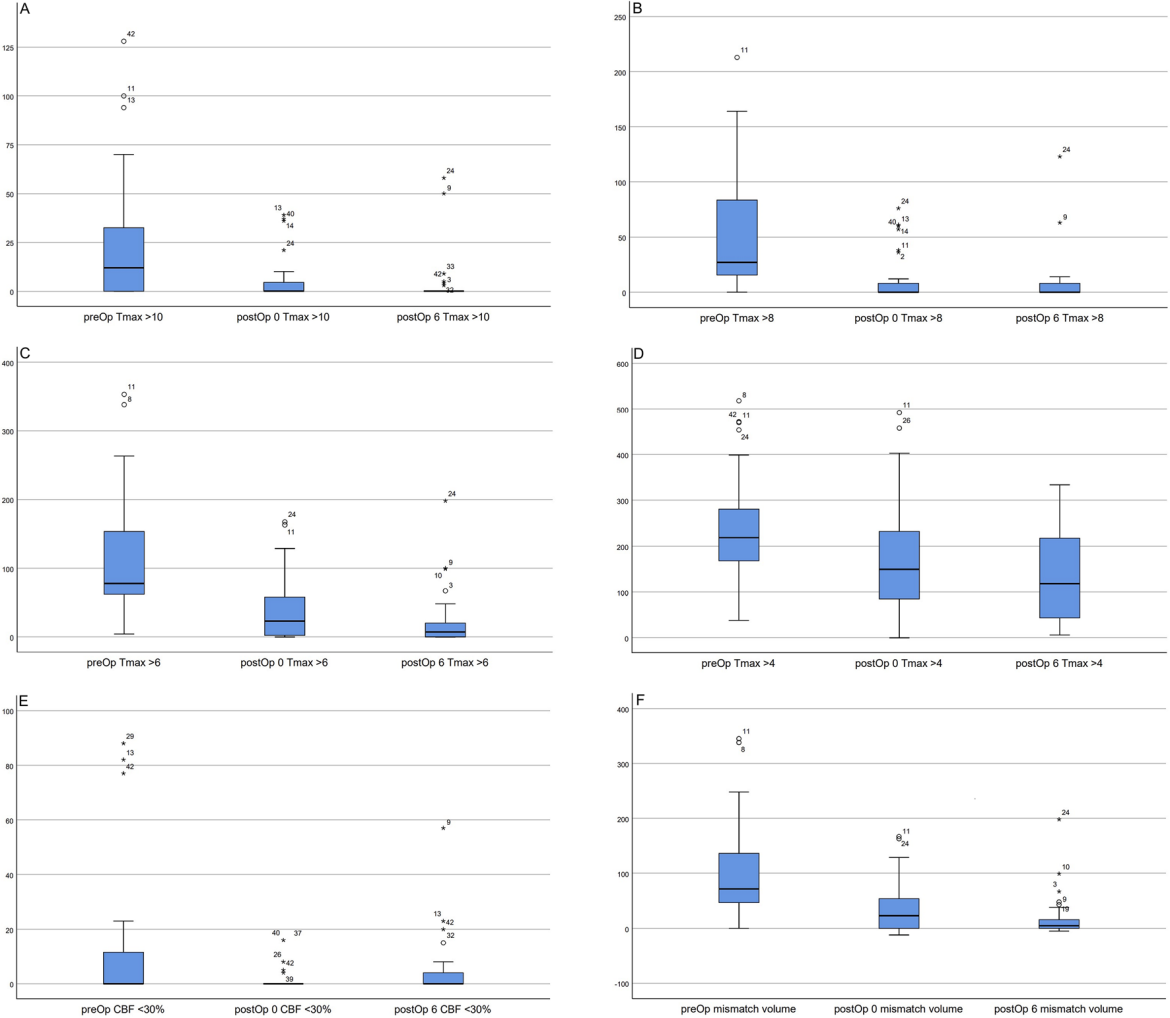
(**A**) The value of Tmax > 10 s of pre-, immediate postoperative, and 6 months postoperative presented a continuous decrease as follows: preoperative, 12 mL (0–34); immediate post-operative, 0 mL (0–6); and 6 months postoperative, 0 mL (0–0) (*p* = 0.000). (**B**) The value of Tmax > 8 s of pre-, immediate postoperative, and 6 months postoperative also continued to decrease as follows: preoperative, 27 ml (15–89); immediate postoperative, 0 ml (0–9); and postoperative 6 months, 0 ml (0–8) (*p* = 0.000). (**C**) The value of Tmax > 6 s of pre-, immediate postoperative, and 6 months postoperative also showed statistically significant decrease as follows: preoperative, 78 mL (61–159); immediate postoperative, 23 mL (0–65); and postoperative 6 months, 7 mL (0–22) (*p* = 0.000). (**D**) The Tmax > 4 s of pre-, immediate postoperative, and 6 months postoperative value decreased continuously as follows: preoperative, 219 mL (164–281); immediate postoperative, 150 mL (79–236); and postoperative 6 months, 118 mL (43–223) (*p* = 0.000). (**E**) The CBF < 30% value showed following trend: preoperative, 0 mL (0–12); immediate postoperative, 0 mL (0–0); and 6 months postoperative, 0 mL (0–5) (*p* = 0.023). (**F**) The mismatch volume calculated as the difference between Tmax > 6 s and CBF < 30% continuously decreased as follows: preoperative, 72 mL (47–148); immediate postoperative, 23 mL (0–57); and 6 months postoperative, 5 mL (0–17) (*p* = 0.000).

We performed a follow-up perfusion examination for 31 of our patients at 6 months postoperatively and analyzed the results. The value of Tmax > 10 s showed a continuous decreasing trend as follows: preoperative, 12 ml (0–34); immediate postoperative, 0 ml (0–6); and postoperative 6 months, 0 ml (0–0), which was statistically significant (*p* = 0.000). Tmax > 8 s also continued to decrease as follows: preoperative, 27 ml (15–89); immediate postoperative, 0 ml (0–9); and postoperative 6 months, 0 ml (0–8) (*p* = 0.000). Tmax > 6 s (preoperative, 78 ml [61–159]; immediate postoperative, 23 ml [0–65]; and postoperative 6 months, 7 ml [0–22]; *p* = 0.000) and Tmax > 4 s (preoperative, 219 ml [164–281; immediate postoperative, 150 ml [79–236]; and postoperative 6 months, 118 ml [43–223], *p* = 0.000) also showed statistically significant decreases. The CBF < 30% value showed the following trend: preoperative, 0 ml (0–12); immediate postoperative, 0 ml (0–0); and postoperative 6 months, 0 ml (0–5), which was statistically significant (*p* = 0.023). The mismatch volume calculated by the above method was as follows: preoperative, 72 ml (47–148); immediate postoperative, 23 ml (0–57); and postoperative 6 months, 5 ml (0–17), showing a statistically significant decrease (*p* = 0.000) (Table 2). Figure 2 shows these figures as a graph.

#### Transfemoral cerebral angiography

All patients showed good bypass patency on TFCA performed one week and six months after the operation, and it was confirmed that the flow spread well to intracranial vessels through the STA. No occlusion or stenosis site was recanalized.

### Clinical outcomes

**Figure 3:**
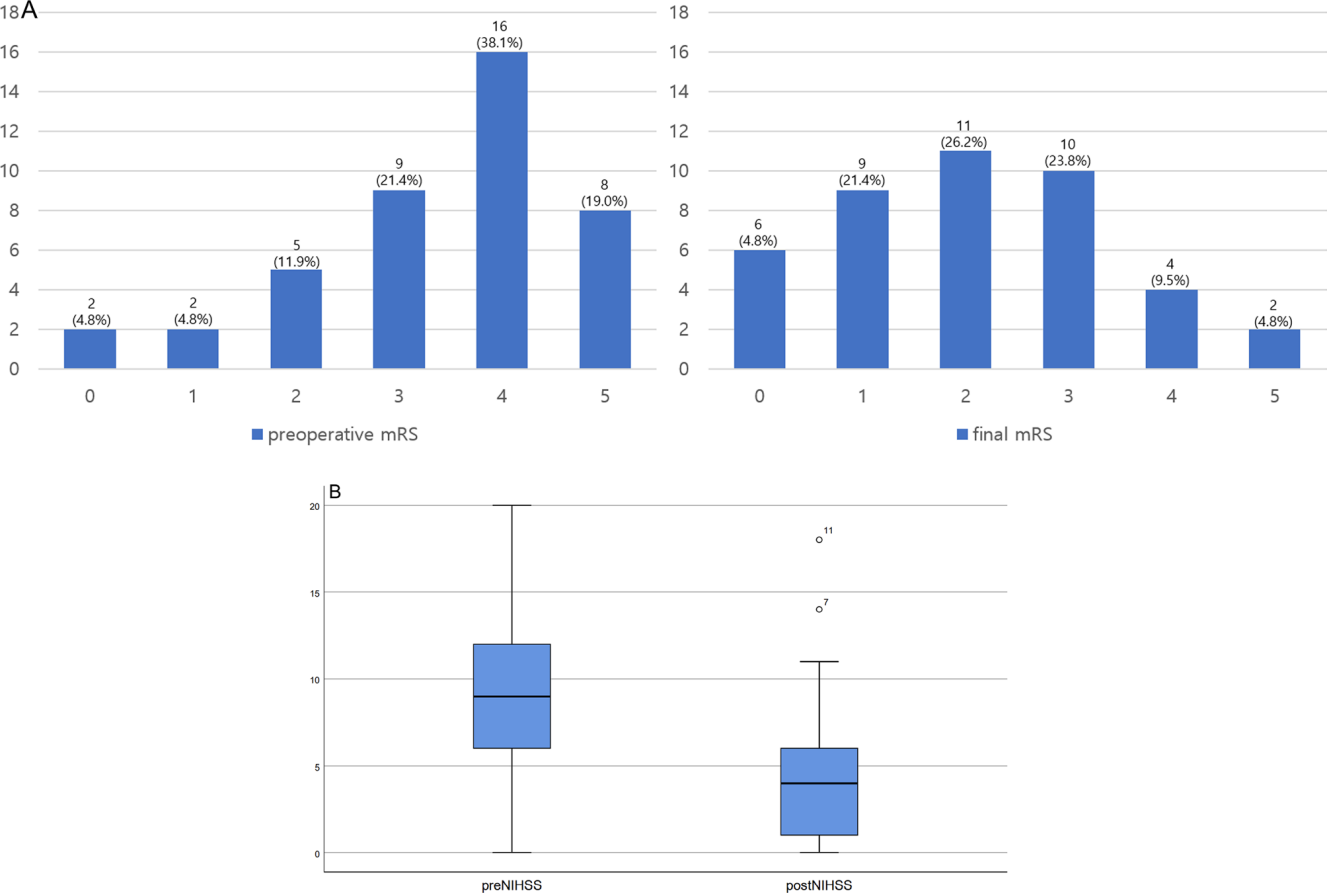
(**A**) The change in mRS before the surgery and mRS at the last follow-up. (**B**) The preoperative NIHSS and NIHSS at discharge demonstrated a significant improvement from 9 (5.75–12) to 4 (1–6.25) (*p* = 0.000).

We followed up patients for 11.7 months (3.85–20.98). Figure 3 show the changes in mRS before the surgery and mRS at the last follow-up. There were 27 patients (64.3%) with mRS of 2 or less, which can be called a good outcome. This is an increase compared to 9 patients (21.4%) preoperatively. Comparing the preoperative NIHSS and the NIHSS at discharge, it was found that there was a significant improvement from 9 (5.75–12) to 4 (1–6.25) (*p* = 0.000).

### Outcome analysis

**Figure 4:**
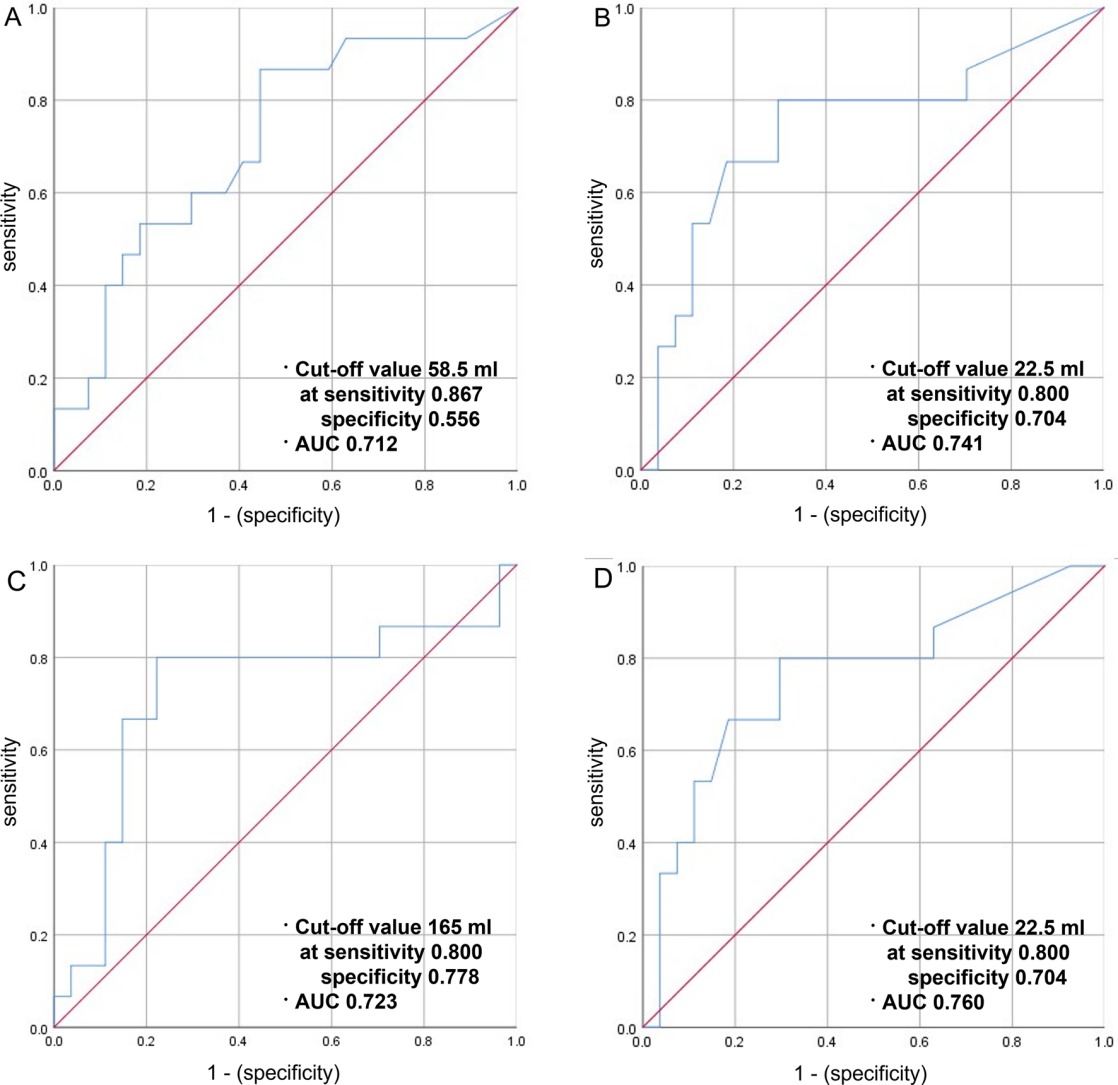
(**A**) The preoperative mismatch volume was observed to be significantly different in the positive and negative outcome group (*p* = 0.044). The AUC was 0.712 and the cut-off value was 58.5 mL. (**B**) The immediate postoperative Tmax > 6 s showed significant difference in the positive and negative outcome group (*p* = 0.027). The AUC was 0.741 and the cut-off value was 22.5 mL. (**C**) The immediate postoperative Tmax > 4 s in the positive and negative outcome group was significantly different (*p* = 0.039), and the AUC and the cut-off value swere 0.723 and 165 ml, respectively. (**D**) The immediate postoperative mismatch volume in the positive and the negative outcome group presented significant difference (*p* = 0.020). The AUC and the cut-off values were 0.760 and 22.5 ml, respectively.

In univariable analysis, preoperative mRS and preoperative NIHSS were found as preoperative factors that influence the good postoperative outcome. Although preoperative CTP parameter was not a factor influencing good outcomes, the volume of immediately postoperative Tmax > 6 s (*p* = 0.029) was revealed to be significant. Nevertheless, multivariable analysis revealed that only preoperative mRS and preoperative NIHSS were essential predictive factors (Table 3). Among all preoperative factors, there were only a few definite factors affecting the postoperative outcome of patients, so we performed Mann–Whitney U-test and chi-square test by dividing the patients into those with good and bad outcomes to find out if there were any significantly different CTP parameters or other variables between the two groups (Table 1). As a result, preoperative mismatch volume (*p* = 0.044), immediately postoperative Tmax > 6 s (*p* = 0.027), Tmax > 4 s (*p* = 0.039) and mismatch volume (*p* = 0.020) were found to be significantly different in the good and bad outcome groups. We identified the area under the curve (AUC) by drawing the receiver operating characteristic curve for the three parameters (Fig. 4). The AUC was 0.712, 0.741, 0.723, and 0.760, and the cut-off values were 58.5 ml, 22.5 ml, 165 ml, and 22.5 ml, respectively.

**Table 3:** Univariable and multivariable analysis of factors affecting good outcome.

Variables	Univariable analysis		Multivariable analysis	
	OR (95% CI)	*p* value	OR (95% CI)	*p* value
Age	0.937 (0.855–1.027)	0.107		
Sex (female)	0.727 (0.180–2.939)	0.655		
Side (right)	1.031 (0.285–3.735)	0.963		
**Medical history**				
Hypertension	1.333 (0.361–4.926)	0.666		
Diabetes mellitus	1.000 (0.262–3.815)	0.999		
Hyperlipidemia	0.455 (0.115–1.795)	0.260		
Onset to operation	0.918 (0.801–1.052)	0.577		
**TOAST classification**				
LAA	Reference	–		
Cardioembolism	1.917 (0.239—15.35)	0.540		
Others	2.000 (0.090–44.35)	0.661		
**Location of lesion**				
Proximal ICA	Reference	–		
Distal ICA	2.000 (0.153–26.19)	0.597		
M1	8.000 (0.310–206.37)	0.210		
M2	6.667 (0.437–101.73)	0.172		
**Lesion severity**				
Occlusion	Reference	–		
Severe stenosis	1.273 (0.105–15.39)	0.999		
**Number of anastomosis**				
Single barrel	Reference	–		
Double barrel	0.677 (0.114–4.004)	0.221		
PreOp mRS	0.126 (0.030–0.525)	0.010	0.535 (0.256–1.118)	0.036
PreOpNIHSS	0.550 (0.375–0.806)	0.001	0.524 (0.246–1.117)	0.019
**PreOp RAPID**				
Tmax > 10 s	0.992 (0.975–1.010)	0.070	0.990 (0.893–1.097)	0.845
Tmax > 8 s	0.990 (0.979–1.002)	0.070	0.994 (0.943–1.046)	0.808
Tmax > 6 s	0.993 (0.986–1.000)	0.150		
Tmax > 4 s	0.997 (0.992–1.001)	0.297		
CBF < 30%	1.008 (0.977–1.040)	0.123		
Mismatch volume	0.992 (0.985–1.000)	0.289		
**PostOp 0 RAPID**				
Tmax > 10 s	0.960 (0.901–1.022)	0.174		
Tmax > 8 s	0.978 (0.947–1.011)	0.190		
Tmax > 6 s	0.981 (0.964–0.998)	0.029	80.418 (0.000–)	0.998
Tmax > 4 s	0.994 (0.989–1.000)	0.052	0.997 (0.984–1.009)	0.998
CBF < 30%	82.710 (0.000–)	0.998		
Mismatch volume	0.979 (0.962–.997)	0.023	0.012 (0.000–)	0.998

CBF, cerebral blood flow; CI, confidence interval; mRS, ICA, internal carotid artery; LAA, large-artery atherosclerosis; modified Rankin Scale; NIHSS, National Institute of Health Stroke Scale; OR, odds ratio; preOp, preoperative; postOp 0, immediate postoperative; s, seconds; Tmax, time-to-maximum; TOAST, Trial of Org 10172 in Acute Stroke Treatment.

### Complications

We analyzed all postoperative complications. Three (7.1%) were cases of silent infarction on diffusion MR performed for routine follow-up without worsening of the symptoms. These 3 patients showed similar clinical course as those without silent infarction. There was one (2.4%) case of infarction progression, even though the patient underwent bypass. On the second postoperative day, the patient complained of worsening weakness on the left side. Multiple new infarctions in the right parietal and temporal cortex were identified on the diffusion MRI. The patient finally recovered just as well as other patients. No patients showed stroke recurrence.

There was one case of postoperative epidural hematoma after double-barrel bypass. After emergent hematoma removal surgery, the patient recovered well. In addition, there was one patient who underwent reoperation due to an infection at the surgical site. No patient died from cerebral complications.

## Discussion

We demonstrated in our previous study that advancing imaging techniques have made it possible to select a target treatment for patients with AIS^10^. After our previous study, various studies have been published to address the effect of EIB. Horiuchi et al. suggested that EIB improves neurological function in approximately 70% of patients^9^. Gunawardena et al. suggested that hemodynamic insufficiency could be a rationale for EIB^7^. A recent meta-analysis of EIB papers demonstrated that EIB may be beneficial for IAT-ineligible patients^6,10,15–23^. Unlike earlier studies, recent research on surgical reperfusion in patients with AIS has produced promising results.

We believe the different results of earlier and more recent studies could result from different patient selection and operation criteria as there has been skepticism for these features in EIBT and COSS studies^24–28^. Furthermore, surgical tools and skills have advanced, leading to improved outcomes in recent years. It is thus reasonable to re-evaluate the role of EIB in the treatment of AIS with large vessel occlusion.

In recent years in particular, various studies have been focused on perfusion examination using CT and MRI as well as the importance of each parameter. In 2020, the American Heart Association scrutinized research on the interpretation of perfusion studies in clinical practice^14^. They reviewed the literature to examine the validity of salvageable tissue or penumbra assessment. They also argued that as perfusion imaging techniques develop, it is possible to identify patients who will benefit from reperfusion from among patients who are beyond the conventional timeframe. Furthermore, as the collateral channel can be different for each patient, they also insisted that individualized treatment is possible when using a perfusion examination rather than simply taking the time from symptom onset.

In our study, some areas of hypoperfusion due to large vessel occlusion had already become ischemic cores, but there are cases where a significant area still had salvageable tissue, even after 24 h, which is the IAT timeframe. The reason for symptom fluctuation despite the treatment for induced hypertension when IAT fails is probably because the perfusion state in the penumbra region waxes and wanes. There have been studies showing that the patients’ neurological status is improved after EIB, but this is the first study to quantitatively prove that the perfusion state improves when EIB is performed in these cases.

Furthermore, immediately after EIB, the Tmax was significantly improved in all sections in this study. Assuming that the part of Tmax > 6 s minus CBF < 30% is the penumbra, the size of the penumbra also significantly decreased pre- and postoperatively. These changes occurred between an examination performed at 24 h preoperatively and 48 h postoperatively; therefore, it is reasonable to assume that these changes were due to surgery rather than natural course of collateral vessel formation after cerebral infarction. Considering that there was no case of spontaneous reopening of occlusion or stenosis in TFCA performed 1 week postoperatively, it is more reasonable to argue that this change is due to EIB. Notably, CBF < 30%, known to represent an ischemic core in several studies, have been shown to decrease slightly immediately postoperatively in our study. This is considered to be because in the acute ischemic state, cerebral edema occurs around the ischemic core; due to this, CBF < 30% appeared in a wider range than the actual ischemic core.

Only the preoperative neurological condition of the patient was found as the preoperative factor predicting good outcome. Among the CTP parameters, there was no factor that could predict good postoperative outcomes. This could be due to selection bias as this study includes patients who have been selected using strict operation criteria. Instead, in the postoperative comparison between the good and bad outcome groups, preoperative mismatch volume, immediate postoperative Tmax > 6 s, Tmax > 4 s, and mismatch volume were significantly different. This means that the patient with a preoperative mismatch volume of < 58.5 ml should undergo surgery, and the patient's mismatch volume must be clearly improved to < 22.5 ml immediately postoperatively to achieve a good outcome. In other words, it is important that an experienced surgeon performs the surgery skillfully to obtain good prognosis. In all patients in our study, STA patency was well maintained in TFCA at postoperative 1 week. The high patency may be attributed to the difference in characteristics of the disease itself partly in light of the many experiences of moyamoya disease treatment at our hospital, but basically, the patient's perfusion status improves a lot when the bypass is well done.

Among the patients in this study, despite three silent infarctions, one infarction progression, and one postoperative hematoma, none of the patients showed postoperative deterioration. The infarction progression rate was 2.56%, similar to recent studies^6,9,17^. The percentage of patients with positive postoperative outcomes was 61.5%, similar to other studies findings^7,16^. Importantly, none of the patient samples of the current study experienced hyperperfusional hemorrhage. Compared to previous studies, wherein the incidence of intracerebral hemorrhage (ICH) after IAT was approximately 5%, the incidence of hyperperfusional hemorrhage in this study was still relatively low despite the operation being performed beyond the accepted timeframe from symptom onset. However, the target demographic of previous studies was different from ours; therefore, the comparison may not be valid. Nevertheless, since antegrade recanalization allows a high flow to the ischemic core, hyperperfusional ICH may be reduced by performing retrograde recanalization, with lower flow, by STA-MCA bypass of the blood vessels, avoiding the ischemic core.

This study was conducted with larger number of patients than previous studies; however, there are still a few limitations. The number of patients is not absolutely large. In addition, it has a disadvantage of being a retrospective study without a control group. Furthermore, we failed to predict what factors should be preoperatively considered to make prognosis good. Further randomized controlled studies with a larger number of patients are warranted to judge whether urgent EIB is actually effective in IAT-ineligible patients with AIS due to large vessel occlusion.

## Conclusion

For carefully selected IAT-ineligible hemodynamic compromised AIS with large vessel occlusion patients, emergent EIB can be an alternative treatment that can improve not only the patient’s perfusion status quantitatively radiologically but also the patient’s neurological status clinically. The immediate postoperative perfusion status is correlated to the good outcome.

## Supplementary Information

Supplementary Information.

## Abbreviations

AIS: Acute ischemic stroke
IAT: Intra-arterial thrombectomy
EC-IC: Extracranial-to-intracranial
EIB: EC-IC bypass
CTP: Perfusion computed tomography
Tmax: Time-to-max
STA-MCA: Superficial temporal artery-to-middle cerebral artery
TTP: Time-to-peak
CBF: Cerebral blood flow
mRS: Modified Rankin Scale
NIHSS: National Institutes of Health Stroke Scale
