# Supplementary material for: Quantitative radiological analysis and clinical outcomes of urgent EC-IC bypass for hemodynamic compromised patients with acute ischemic stroke
Source: Sci Rep. 2022 May 25;12:8816. doi: 10.1038/s41598-022-12728-x (PMC9132915; doi:10.1038/s41598-022-12728-x)

Quantitative Radiological Analysis and Clinical Outcomes of Urgent EC-IC Bypass for Hemodynamic Compromised Patients with Acute Ischemic Stroke

Hyunjun Jo, MD[1], Dongwook Seo, MD[2], Young-Deok Kim, MD[2], Seung Pil Ban, MD[2], Tackeun Kim, MD[2], Oki Kwon, MD, PhD[2], Chang Wan Oh, MD, PhD[2], Leonard Sunwoo, MD, PhD[3], Beom Joon Kim, MD, PhD[4], Moon-Ku Han, MD, PhD[4], Hee-Joon Bae, MD, PhD[4], Si Un Lee, MD[2], and Jae Seung Bang, MD[2]

[1]Department of Neurosurgery, Korea University Ansan Hospital, Korea University College of Medicine, Ansan, Korea

[2]Department of Neurosurgery, Seoul National University Bundang Hospital, Seoul National University College of Medicine, Seongnam-si, Korea

[3]Department of Radiology, Seoul National University Bundang Hospital, Seoul National University College of Medicine, Seongnam-si, Korea

[4]Department of Neurology, Seoul National University Bundang Hospital, Seoul National University College of Medicine, Seongnam-si, Korea

Address the correspondence to

Jae Seung Bang

Department of Neurosurgery, Seoul National University Bundang Hospital,

Seoul National University College of Medicine

82 Gumi-ro 173 beon-gil, Bundang-gu, Seongnam-si, Gyeonggi-do, 13620, Korea

Tel: 82-31-787-7172

Fax: 82-31-787-4059

E-mail: nsbang@snubh.org

Si Un Lee

Department of Neurosurgery, Seoul National University Bundang Hospital,

Seoul National University College of Medicine

82 Gumi-ro 173 beon-gil, Bundang-gu, Seongnam-si, Gyeonggi-do, 13620, Korea

Tel: +82-31-787-7169

Fax: +82-31-787-4059

E-mail: nsmidget@gmail.com

Running title

Urgent bypass for acute ischemic stroke

Supplementary Figure S1

Figure S1A and S1B:

A 62-year-old visited the emergency room with moderate weakness on the right hand that occurred 1 hour and 30 minutes ago, and the NIHSS score was 6 points. On MRI, scattered infarction was found in the left parietal lobe and the occlusion in the inferior division of the left M2 was diagnosed on transfemoral carotid angiography (TFCA).

Figure S1C:

RAPID map on perfusion CT showed that the volume of Tmax >6seconds was 90ml.

Figure S1D:

Mechanical thrombectomy and intra-arterial thrombolysis were attempted, but failed and it worsened to NIHSS 8, and emergency superficial temporal artery-M4 bypass was performed.

Figure S1E and S1F:

After the operation, TFCA (fig. S1E, AP view; fig. S1F, lateral view) demonstrated abundant flow of bypass which supplied the territory of left inferior M2.

Figure S1G:

The patient's symptoms recovered, and the perfusion delay was significantly improved in the perfusion CT performed immediately after the operation.

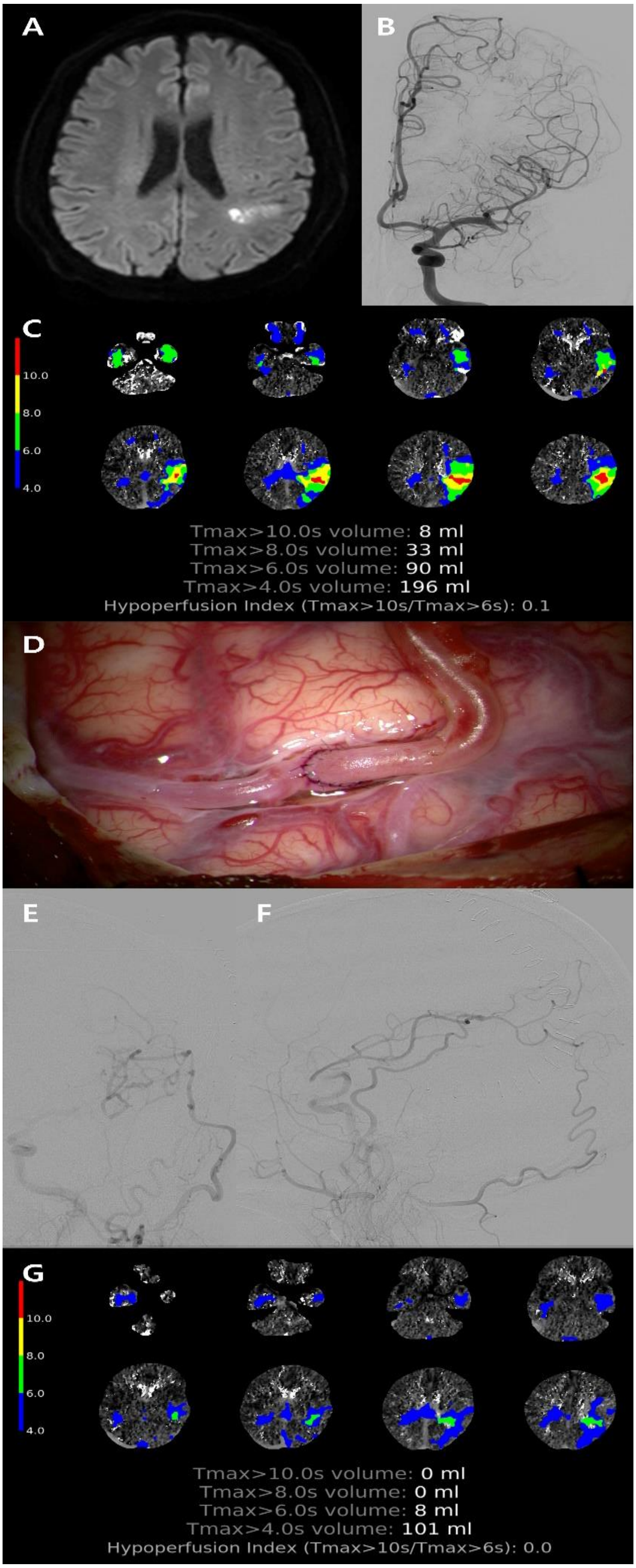

Supplementary Figure S2

Figure S2A and S2B:

A 65-year-old male patient presented motor transient ischemic attack on the right side 4 days before admission. Right hemiparesis worsened 2 days after symptom onset and did not recover, thus he visited the emergency room with a NIHSS score of 7. Left paramedian border-zone infarction was identified on MRI and transfemoral carotid angiography (TFCA) showed left proximal internal carotid artery occlusion.

Figure S2C:

RAPID map on perfusion CT showed that the volume of Tmax >6seconds was 75ml.

Figure S2D:

The endovascular treatment was impossible because 2 days had passed since the onset of symptoms and the possibility of chronic type of occlusion was highly suspected in MRA. In addition, perfusion delay was severe on perfusion CT and fluctuation in symptoms according to blood pressure was much frequent, so emergency bypass has been performed.

Figure S2E and S2F:

After the operation, TFCA (fig. S2E, AP view; fig. S2F, lateral view demonstrated abundant flow of bypass which supplied most of left MCA territory.

Figure S2G:

The patients improved to NIHSS score of 4, and perfusion delay was much improved.

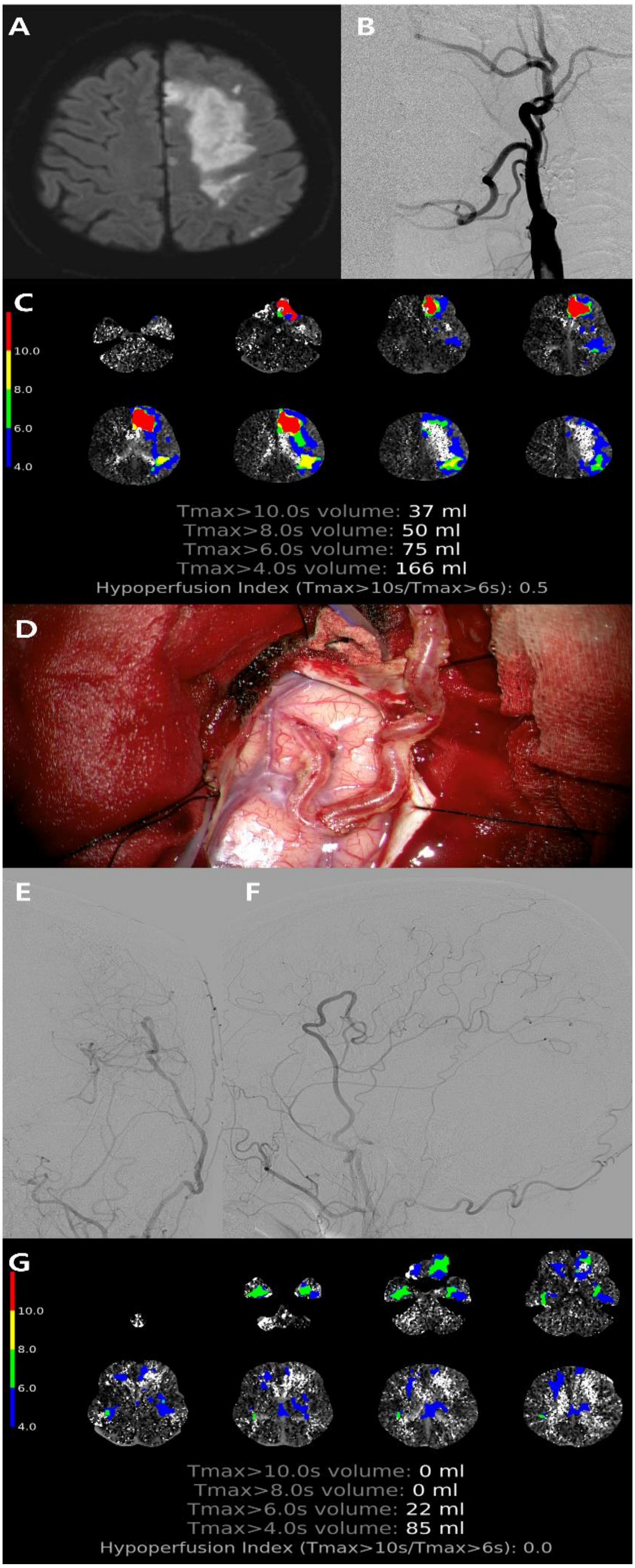

Supplement: Supplementary file 1 — Supplementary Information. [file 41598_2022_12728_MOESM1_ESM.pdf]
